# Multimodal data integration for enhanced longitudinal prediction for cardiac and cerebrovascular events following initial diagnosis of obstructive sleep apnea syndrome

**DOI:** 10.7189/jgh.14.04103

**Published:** 2024-05-17

**Authors:** Tong Zhou, Yijun Wang, Yanan Xu, Li Xu, Long Tang, Yi Yang, Jun Wang

**Affiliations:** 1 Department of Cardiology, First Affiliated Hospital of Bengbu Medical University, Bengbu, China; 2 Department of Cardiology, Renmin Hospital of Wuhan University, Wuhan, China; 3 Pulmonary and Critical Care Medicine, First Affiliated Hospital of Bengbu Medical University, Bengbu, China; 4 Department of Cardiology, Guiqian International General Hospital, Guiyang, China; 5 Department of Cardiology, People’s Hospital of Xuancheng City, Affiliated Xuancheng Hospital of Wannan Medical College, Xuancheng, China; 6 Department of Cardiology, Xinjiang Medical University, Urumqi, China; 7 Department of Cardiology Fourth Ward, Xinjiang Medical University Affiliated Hospital of Traditional Chinese Medicine, Urumqi, China

## Abstract

**Background:**

Obstructive sleep apnea syndrome (OSAS), a prevalent condition, often coexists with intricate metabolic issues and is frequently associated with negative cardiovascular outcomes. We developed a longitudinal prediction model integrating multimodal data for cardiovascular risk stratification of patients with an initial diagnosis of OSAS.

**Methods:**

We reviewed the data of patients with new-onset OSAS who underwent diagnostic polysomnography between 2018–19. Patients were treated using standard treatment regimens according to clinical practice guidelines.

**Results:**

Over a median follow-up of 32 months, 98/729 participants (13.4%) experienced our composite outcome. At a ratio of 7:3, cases were randomly divided into development (n = 510) and validation (n = 219) cohorts. A prediction nomogram was created using six clinical factors – sex, age, diabetes mellitus, history of coronary artery disease, triglyceride-glucose index, and apnea-hypopnea index. The prediction nomogram showed excellent discriminatory power, based on Harrell’s C-index values of 0.826 (95% confidence interval (CI) = 0.779–0.873) for the development cohort and 0.877 (95% CI = 0.824–0.93) for the validation cohort. Moreover, comparing the predicted and observed major adverse cardiac and cerebrovascular events in both development and validation cohorts indicated that the prediction nomogram was well-calibrated. Decision curve analysis demonstrated the good clinical applicability of the prediction nomogram.

**Conclusions:**

Our findings demonstrated the construction of an innovative visualisation tool that utilises various types of data to predict poor outcomes in Chinese patients diagnosed with OSAS, providing accurate and personalised therapy.

**Registration:**

Chinese Clinical Trial Registry ChiCTR2300075727.

Obstructive sleep apnea syndrome (OSAS) is a serious health condition that can negatively impact individuals’ overall well-being, especially accelerated atherosclerosis and increased cardiovascular disease risk [1–3]. Early detection and intervention of OSAS have been shown to significantly improve clinical outcomes and enhance sleep-related quality of life [1–3]. Thus, the promotion of public awareness regarding OSAS and the evaluation of individuals’ ability to assess the risk of adverse events in OSAS patients are crucial for encouraging appropriate medical attention-seeking behaviour, thereby preventing or managing the associated detrimental consequences. Notably, significant efforts are being made in well-resourced settings to diagnose and treat individuals with OSAS. However, the available data suggest that most cases of OSAS remain undiagnosed and untreated [1–4]. In China, there is generally a lack of awareness regarding OSAS, and diagnostic and treatment options are often unavailable or not adapted for resource-poor settings [1]. Multiple studies have demonstrated that continuous positive airway pressure (CPAP) treatment for moderate-to-severe OSAS with coexisting cardiovascular disease offers little benefit in terms of cardiovascular risk reduction [5,6]. Therefore, strategies for early prediction of OSAS and risk stratification are needed to address this health problem and prevent irreversible harm to individuals and society.

OSAS advances through an intricate and evolving process that can result in serious complications and is associated with various immunometabolic disorders, increasing the risk of cardiovascular events [1–4,7]. Accordingly, a one-size-fits-all approach to the diagnosis and management of OSAS does not reflect the complexity of the disease. It cannot quantify the severity or risks of cardiovascular events for individual cases. Thus, a growing body of research aims to identify reliable markers for the severity of OSAS and the risks of adverse outcomes [7–11]. The available data supports a transition from focusing on multiple dimensions of a multifactorial response network to understanding OSAS as a metabolic disorder affected by various systemic factors [11]. The availability of novel statistical methods has allowed the construction of visual risk stratification tools incorporating multimodal data sets, with a putative predictive factor in each set [12–16]. Data from a decade-long historical cohort study of 10 149 patients diagnosed with or referred for OSAS found that patients’ demographic and clinical features combined with physiologic indices provided better prognostic value for predicting cardiovascular events and all-cause mortality. However, this study did not include validation cohorts or establish the clinical applicability of their prognostic model [15]. Additionally, the previous model did not consider inflammatory, metabolic, or other important categories of biomarkers.

To the best of our knowledge, no studies have investigated the value of multimodal data (clinical examination data, biomarker, and sleep monitoring data) integration for longitudinal prediction of the initial diagnosis of OSAS in a Chinese population. The purpose of the present study was to develop a predictive model for prognosis in patients with a new diagnosis of OSAS who were treated using standard treatment regimens among a Chinese population based on multimodal data of factors that influence outcomes in these patients.

## METHODS

### Study design and participants

Our study used data from a cohort study (OSAS patients, n = 729) from January 2018 to December 2019. We included the data from all consecutive patients with OSAS who underwent a successful polysomnography examination for initial diagnosis at the Xinjiang Medical University Affiliated Hospital of Traditional Chinese Medicine. The study time frame from 2018–19 represents the most up-to-date data available at the study initiation, ensuring that the findings accurately reflect current clinical practices and patient characteristics. Moreover, the chosen timeframe aligns with the publication of significant guidelines and consensus statements on the diagnosis and management of OSAS, including the 2017 American Academy of Sleep Medicine clinical practice guidelines [17]. These guidelines provided updated recommendations for diagnostic criteria, treatment protocols, and follow-up strategies, potentially influencing clinical decision-making and patient outcomes during the study period [17]. By focusing on the years 2018–19, our objective was to examine the impact of these recent guidelines on real-world clinical practice and ensure the relevance of our findings in relation to the current landscape of OSAS management. All eligible cases were randomly assigned to the development (n = 510) and validation (n = 219) cohorts. Table S1 in the **Online Supplementary Document** contains a thorough list of the criteria for including and excluding participants in this research. Figure S1 in the **Online Supplementary Document** presents a flowchart of the study procedure.

The study was approved by the institutional review board of Xinjiang Medical University Affiliated Hospital of Traditional Chinese Medicine (number 2022XE0103-1). Because this was a retrospective study, the institutional review board waived the requirement for informed consent from patients.

### Sleep monitoring

The Grael system from Compumedics (Melbourne, Australia) was utilised to conduct a comprehensive overnight laboratory-based polysomnograph for each eligible participant. The staff members responsible for analysing the polysomnography data were explicitly blinded to the patient’s clinical status, ensuring they remained unaware of any clinical diagnoses, treatments, or outcomes associated with the patients from whom the polysomnography data were obtained. The sleep-related variables were derived from sleep monitoring, such as lowest arterial oxygen saturation, lowest heart rate during sleep, highest heart rate during sleep, and mean heart rate. A diagnosis of apnea was made when airflow dropped by 90% from the pre-event baseline for at least 10 seconds.

Hypopnea was identified as a reduction in airflow of at least 30% compared to the baseline for a minimum of 10 seconds, along with a decrease in oxygen saturation of at least 4% or a decrease in airflow of at least 50% lasting for at least 10 seconds as well as a decrease in oxygen saturation of at least 3%. To determine the frequency of apnea and hypopnea events per hour of sleep, the apnea-hypopnea index (AHI) was measured. The recorded data was manually scored by two experienced sleep technicians in accordance with the 2017 American Academy of Sleep Medicine Clinical Practice Guideline [17,18]. Before sleep monitoring, the neck circumference, abdominal girth, weight, and height were measured for each patient. Body mass index (BMI) was calculated as weight in kilograms divided by height in square meters. Following discharge, patients received treatment in accordance with clinical practice guidelines [16], which encompassed lifestyle modifications such as weight reduction, abstinence from alcohol consumption, promotion of regular sleep patterns, and utilisation of CPAP as the primary therapeutic approach for moderate and severe cases.

### Laboratory tests

The morning following admission, a fasting blood sample was collected from a peripheral vein and tested for biomarkers such as fasting glucose, haemoglobin A1c (HbA1c), lipids, fibrinogen, and D-dimer levels, along with kidney function biomarkers and standard blood cell counts.The fibrinogen level, white blood cell count, neutrophil-to-lymphocyte ratio, and platelet-to-lymphocyte ratio were identified as inflammation biomarkers. Furthermore, the triglyceride-glucose index (TyG) index was determined to assess insulin resistance by calculating natural logarithm (fasting triglyceride (mg/dl) × fasting plasma glucose (mg/dl)/2) [19].

### Clinical endpoint

We conducted follow-up visits or telephone interviews to monitor patients for the occurrence of our primary clinical endpoint and major adverse cardiac and cerebrovascular events (MACCEs). The diagnosis of each event was determined according to well-established clinical criteria and evaluated by an independent expert committee that remained unaware of the participant’s group assignment. We conducted thorough assessments at every visit, encompassing physical examinations, medical record reviews, and adverse event evaluations. To maintain methodological consistency, a standardised questionnaire was employed for data collection during both face-to-face visits and telephone interviews. All participant-reported events underwent rigorous verification through meticulous scrutiny of medical records followed by evaluation from an unbiased committee. The average duration of follow-up was 30.65 months for the study population. Various serious cardiac events, including cardiac mortality, acute coronary syndrome, and stroke, were considered MACCEs.

### Constructing the predictive model

We have presented a comprehensive inventory of the multimodal data incorporated in our models, meticulously selected based on their clinical relevance and potential impact on MACCEs. The variables encompassed demographic characteristics such as age and sex, clinical factors including BMI, smoking status, alcohol consumption status, comorbidities, laboratory parameters, and sleep-related variables. We utilised the createDataPartition function from the caret package to perform the splitting of the machine learning data set into development and validation cohorts, with a designated train-test ratio of 7:3. To address the problem of overfitting, we incorporated a penalty parameter, also known as a tuning parameter, into the adaptive least absolute shrinkage and selection operator (Lasso) regression model. This parameter was utilised to penalise the coefficients of variables included in the model, thereby mitigating the risk of overfitting [20]. Moreover, this approach enables the identification of the most relevant predictors from a potentially extensive pool of variables, which proves especially valuable in our study involving multiple clinical variables [20]. In the development cohort, potential predictors were screened using Lasso regression with 10-fold cross-validation. Subsequently, the prognostic model of independent factors for MACCEs was constructed based on factors identified using multivariate Cox’s proportional hazards model, which allowed for calculating hazard ratios (HRs) with 95% confidence intervals (CIs).

The performance of the prognostic model was evaluated using established criteria [12,21]. Using receiver operating characteristic curve analysis, we evaluated the predictive model’s ability to differentiate by computing Harrell’s concordance index (C-index) and its 95% CI. Calibration graphs were created to assess the accuracy of the prognostic model by comparing projected MACCE risk probabilities at two and three years with actual results. The net benefit of the prognostic model was evaluated at various threshold probabilities through decision curve analysis (DCA).

The prognostic and individual models were assessed for net benefit rates across six variables (sex, age, diabetes mellitus, history of coronary artery disease, TyG index, and AHI) in both the development and validation groups. We utilised the predictive algorithm to calculate a personalised risk assessment for every patient in both the development and validation groups, resulting in the identification of low-risk and high-risk categories based on the most effective threshold for each group. Furthermore, the overall MACCE-free survival rate was assessed through the Kaplan-Meier technique, categorising patients into low-risk and high-risk categories based on the outcomes. We utilised a log-rank test to ascertain the variance in MACCE risk levels between the low-risk and high-risk categories.

### Statistical analysis

Statistical analysis was conducted using R, version 4.2.2(R Core Team, Vienna, Austria) and SPSS, version 23 (IBM, Armonk, New York, USA). As descriptive statistics, percentages and frequencies were reported for categorical variables, and median values with interquartile ranges (IQR) or mean values with standard deviations were reported for continuous variables. We assessed non-parametric continuous data with the Mann-Whitney U test, while distinctions in categorical variables were identified through the Fisher exact test or χ^2^ test. Statistical significance was determined for all variances with a *P* < 0.05.

## RESULTS

### Clinical features of the study population

The development and validation cohorts included 510 and 219 eligible patients, respectively. A total of 169 patients underwent CPAP treatment. The median duration from the initial OSAS diagnosis to the first clinical outcome evaluation was 32 months (IQR = 27.0–35.0) for the entire cohort. Comparable rates of MACCEs were observed in the development and validation groups. Additionally, no differences in sleep monitoring data, laboratory test results, and demographic characteristics were detected between the development and validation cohorts (**Table 1**). The utilisation of our data mitigates the potential for bias in evaluating the performance of the predictive model and enhances the generalisability of our findings to similar populations.

**Table 1:** Baseline patient characteristics and MACCEs (clinical outcome) in the development and validation cohorts

Characteristic or outcome	All cohort (n = 729)	Development cohort (n = 510)	Validation cohort (n = 219)	*P*-value
Male sex, n (%)	500 (68.6)	342 (67.1)	158 (72.1)	0.204
Age in years*	54.0 (47.0–61.0)	54.0 (47.0–61.0)	53.0 (47.0–61.0)	0.473
Previous CAD, n (%)	364 (49.9)	255 (50.0)	109 (49.8)	1.000
Current smoking, n (%)	311 (42.7)	210 (41.2)	101 (46.1)	0.248
Current alcohol consumption, n (%)	280 (38.4)	188 (36.9)	92 (42.0)	0.220
Hypertension, n (%)	367 (50.3)	248 (48.6)	119 (54.3)	0.183
Diabetes mellitus, n (%)	140 (19.2)	104 (20.4)	36 (16.4)	0.254
Family history of CAD, n (%)	117 (16.0)	82 (16.1)	35 (16.0)	1.000
Non-alcoholic fatty liver disease, n (%)	400 (54.9)	273 (53.5)	127 (58.0)	0.304
BMI (kg/m^2^)†	28.26 (3.87)	28.15(4.00)	28.55(3.61)	0.196
Abdominal girth (cm)†	94.63 (11.14)	94.24 (11.06)	95.53 (11.32)	0.150
Neck circumference (cm)†	40.83 (4.84)	40.68 (5.10)	41.20 (4.14)	0.151
HbA1c (%)*	5.81 (5.50–6.39)	5.80 (5.49–6.34)	5.85 (5.54–6.43)	0.182
HDL-C (mmol/L)*	1.23 (1.08–1.39)	1.24 (1.08–1.40)	1.20 (1.06–1.37)	0.249
LDL-C (mmol/L)*	2.29 (1.82–2.76)	2.30 (1.84–2.79)	2.25 (1.79–2.74)	0.500
Total cholesterol (mmol/L)*	4.31 (3.65–4.99)	4.34 (3.70–5.01)	4.27 (3.48–4.92)	0.239
Fasting plasma glucose (mmol/L)*	5.25 (4.73–6.15)	5.19 (4.71–6.15)	5.31 (4.79–6.14)	0.334
Triglyceride (mmol/L)*	1.66 (1.21–2.33)	1.65 (1.20–2.30)	1.68 (1.23–2.39)	0.765
TyG*	8.91 (8.51–9.31)	8.91 (8.48–9.31)	8.90 (8.58–9.30)	0.853
Apolipoprotein A1 (g/L)*	1.22 (1.11–1.36)	1.22 (1.11–1.36)	1.22 (1.12–1.35)	0.945
Apolipoprotein B, g/L*	0.92 (0.78–1.08)	0.92 (0.78–1.08)	0.93 (0.78–1.08)	0.810
Lipoprotein (a) (mg/L)*	128 (65.8–260.0)	133 (69.0–249.0)	120 (58.6–282.0)	0.413
Creatinine (mmol/L)*	76.0 (65.0–86.0)	75.5 (64.6–86.0)	76.4 (67.0–87.0)	0.333
Blood urea nitrogen (mmol/L)*	5.30 (4.60–6.20)	5.30 (4.54–6.20)	5.40 (4.60–6.30)	0.374
Uric acid (μmol/L)*	344.0 (295.0–399.0)	340.0 (291.0–394.0)	355.0 (303.0–404.0)	0.061
Fibrinogen (g/L)*	2.94 (2.56–3.41)	2.94 (2.56–3.40)	2.94 (2.59–3.42)	0.820
D-dimer (mg/L)*	0.50 (0.34–0.64)	0.50 (0.32–0.64)	0.51 (0.36–0.65)	0.513
Haemoglobin (g/L)†	147.0 (15.73)	146.95 (14.84)	148.10 (17.66)	0.397
White blood cells (10^9^/L)†	6.69 (1.67)	6.63 (1.70)	6.84 (1.60)	0.122
Neutrophil count (10^9^/L)*	4.21 (3.19–5.61)	4.05 (3.14–5.60)	4.53 (3.51–5.62)	0.064
Monocyte count (10^9^/L)*	2.26 (1.81–2.86)	2.21 (1.73–2.80)	2.39 (1.88–2.96)	0.054
NLR*	1.84 (1.48–2.39)	1.85 (1.46–2.41)	1.80 (1.51–2.34)	0.835
Platelets (10^9^/L)*	235.0 (197.0–272.0)	234.0 (194.0–272.0)	238.0 (200.0–272.0)	0.399
PLR*	104.0 (81.0–133.0)	107.0 (81.1–137.0)	98.8 (80.2–127.0)	0.149
Platelet distribution width (%)†	12.22 (2.09)	12.17 (2.14)	12.33 (1.97)	0.354
Mean platelet volume (fL)†	10.34 (0.97)	10.31 (0.9)	10.40 (0.94)	0.272
Apnea hypopnea index*	15.6 (9.30–28.2)	15.2 (10.0–24.8)	19.4 (7.90–34.7)	0.551
Lowest arterial oxygen saturation*	83.0 (79.0–86.0)	83.0 (80.0–85.0)	83.0 (74.0–89.0)	0.213
Lowest heart rate during sleep (beats/min)*	58.0 (53.0–63.0)	58.0 (52.0–63.0)	59.0 (54.0–64.0)	0.133
Highest heart rate during sleep (beats/min)*	79.0 (73.0–86.0)	78.5 (73.0–85.0)	79.0 (72.0–86.5)	0.595
Mean heart rate (beats/min)*	66.0 (60.0–71.0)	66.0 (60.0–71.0)	66.0 (60.0–71.0)	0.968
MACCEs, n (%)	98 (13.4)	69 (13.5)	29 (13.2)	1.000
Cardiac mortality, n (%)	17 (2.33)	9 (1.76)	8 (3.65)	0.200
Acute coronary syndrome, n (%)	75 (10.3)	54 (10.6)	21 (9.59)	0.784
Stroke, n (%)	23 (3.16)	17 (3.33)	6 (2.74)	0.850

BMI – body mass index, CAD – coronary artery disease, HbA1c – haemoglobin A1c, HDL-C – high-density lipoprotein cholesterol, LDL-C – low-density lipoprotein cholesterol, MACCEs – major adverse cardiac and cerebrovascular events, NLR – neutrophil to lymphocyte ratio, OSAS – obstructive sleep apnea syndrome, PLR – platelet to lymphocyte ratio, TyG – triglyceride-glucose

*Values are presented as medians and interquartile ranges.

†Values are presented as means and standard deviations.

### Prediction nomogram

**Figure 1:**
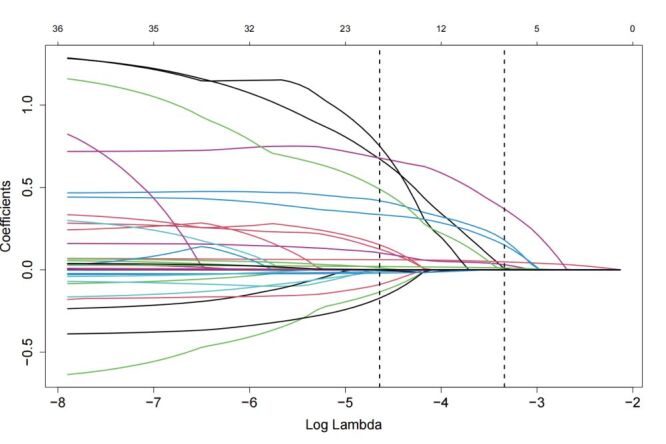
Least absolute shrinkage and selection operator regression plot of the model coefficient trendlines for the 43 variables potentially associated with the risk of MACCEs in obstructive sleep apnea syndrome patients. MACCEs – major adverse cardio and cerebrovascular events

**Figure 2:**
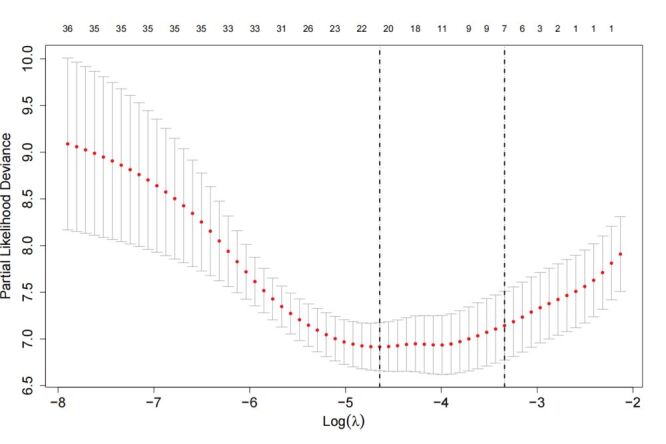
Tuning parameter (λ) selection was performed using a cross-validation error curve with 10-fold cross-validation. Optimal values were identified by the minimum criteria and one standard error criteria, as indicated by vertical lines. The value selected by 10-fold cross-validation was also marked by a vertical line. Seven nonzero coefficients were optimised and selected for further Cox regression analysis.

**Figure 3:**
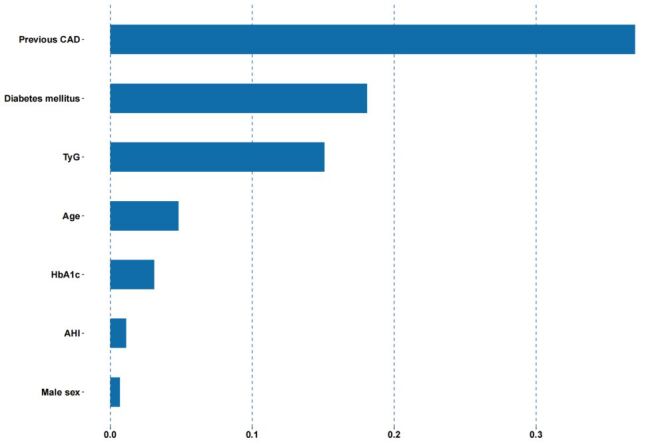
Strength of correlation between variable and MACCEs risk according to correlation coefficients based on varying values of λ one standard error. The y-axis denotes the different variables, while the x-axis represents the magnitude of the correlation coefficient. MACCEs – major adverse cardio and cerebrovascular events

In the Lasso regression, 43 clinical characteristics were included, and the optimal penalisation was determined using 10-fold cross-validation. Seven potential predictors of MACCEs were identified – sex, age, diabetes mellitus, previous coronary artery disease (CAD), TyG index, HbA1c, triglyceride, and AHI (**Figure 1**, **Figure 2**). The correlation coefficients that demonstrate the predictive power of each variable are presented in **Figure 3**.

**Figure 4:**
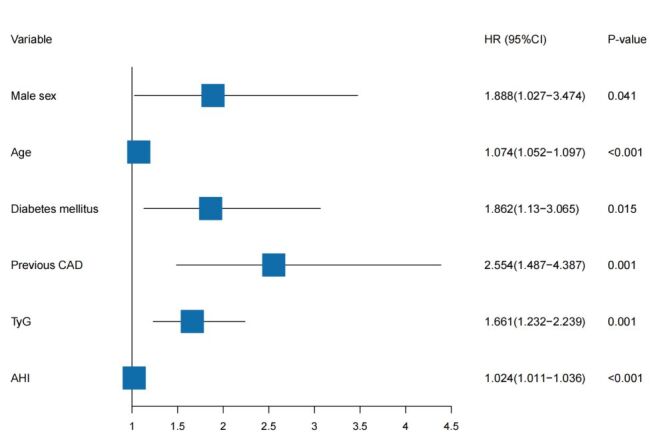
Forest plot with hazard ratios, 95% confidence intervals, and corrected *P*-values for independent prognostic variables identified by multivariate Cox regression analysis.

Further refined analysis using multivariable Cox regression revealed that male sex (HR = 1.888; 95% CI = 1.027 − 3.474, *P* = 0.041), age (HR = 1.074; 95% CI = 1.052 − 1.097, *P* < 0.001), diabetes mellitus (HR = 1.862; 95% CI = 1.13 − 3.065, *P* = 0.015), prior CAD (HR = 2.554; 95% CI = 1.487 − 4.387, *P* = 0.001), TyG index (HR = 1.661; 95% CI = 1.232 − 2.239, *P* = 0.001), and AHI (HR = 1.024; 95% CI = 1.011 − 1.036, *P* < 0.001) were all found to be significant independent predictors of MACCEs in patients with OSAS (**Figure 4**).

**Figure 5:**
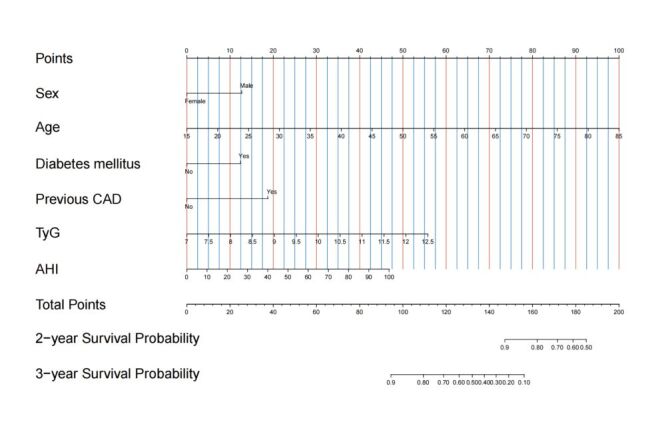
A prognostic nomogram was constructed from the optimal multivariate Cox regression to predict two and three-year survival probabilities from the initial diagnosis of obstructive sleep apnea syndrome in the development cohort.

We then constructed the prediction nomogram by selecting six variables (sex, age, diabetes mellitus, previous CAD, TyG index, and AHI). These were established as predictors significant enough to be included for risk stratification at two and three years (**Figure 5**). Using the nomogram, the two and three-year survival probabilities are calculated by drawing a perpendicular line from the corresponding axis of each predictor to the top line labelled ‘points’. Then, the points are summed for all predictors, and a line is drawn from the axis labelled ‘total points’ to the place where it intercepts each of the survival axes. Accordingly, along this vertical line, the predicted risk corresponding to the ‘total points’ represents the patient’s two and three-year survival probabilities. The corresponding equations for two and three-year survival probability are as follows:

two-year survival probability = 0 × total points^3^ + 0.00013 × total points^2^ + (–0.00199)  × total points +0.39333;

three-year survival probability = 0 × total points^3^ + (–0.00107) × total points^2^ + 0.13293 × total points + (–4.25466).

### Performance of the prognostic nomogram

**Figure 6:**
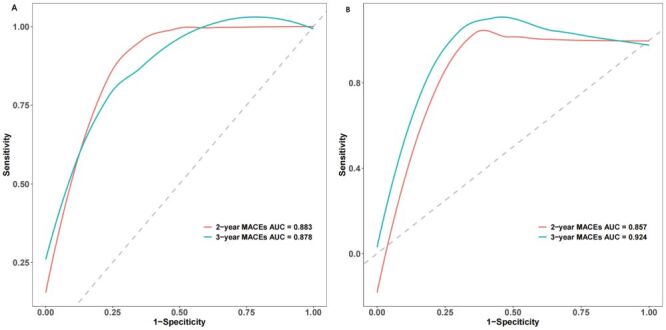
Receiver operating characteristic curve analysis of the discriminative ability of the prognostic nomogram. **Panel A.** Development cohort. **Panel B.** Validation cohort.

**Figure 7:**
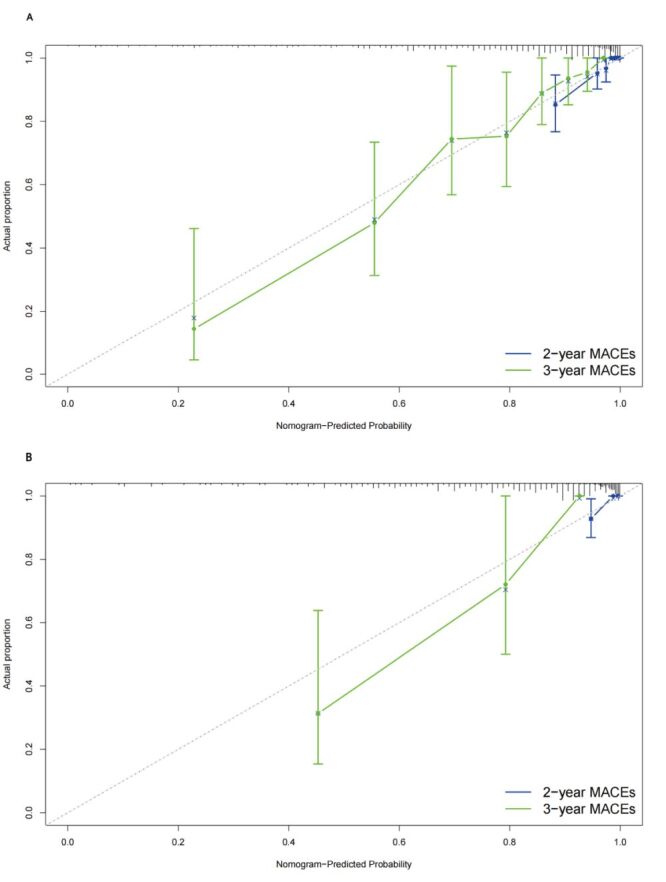
Calibration curves for the prognostic model. **Panel A.** Development cohort. **Panel B.** Validation cohort.

**Figure 8:**
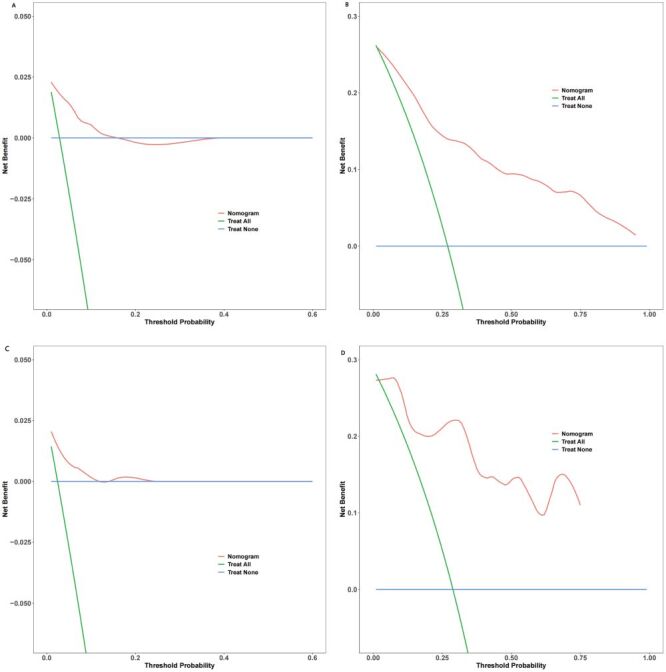
Decision curve analysis of the prognostic nomogram’s ability to predict the risk of MACCEs. **Panel A.** At two years in the development cohort. **Panel B.** At three years in the development cohort. **Panel C.** At two years in the validation cohort. **Panel D.** At three years in the validation cohort. MACCEs – major adverse cardio and cerebrovascular events

Harrell’s C-index and area under the curve (AUC) values were calculated to evaluate the discriminatory ability of the prognostic nomogram to distinguish between MACCEs and without MACCEs outcomes. The prognostic nomogram demonstrated excellent discrimination in separating cases with MACCEs and without MACCEs in both the development and validation cohorts, as indicated by Harrell’s C-index values of 0.826 (95% CI = 0.779–0.873) and 0.877 (95% CI = 0.824–0.930), respectively (**Figure 6****,** panels A–B). This model showed good predictive accuracy for two and three-year MACCEs, with AUC values of 0.883 and 0.878 for two and three-year MACCEs in the development cohort, respectively. Furthermore, in the verification cohort, the nomogram model continued to exhibit good accuracy power, with AUC values of 0.857 and 0.924 for two and three-year MACCEs, respectively. Furthermore, the accuracy of the prognostic nomogram, which measures the deviation of predictions from actual results, was evaluated using a calibration chart. A strong correlation was found between the observed incidence of MACCEs and the predicted likelihood of MACCEs using the nomogram in both the development and validation groups (**Figure 7**, panels A–B). The clinical utility of the prognostic nomogram, which represents its ability to characterise potential decision thresholds, was demonstrated by DCA. In the development cohort, the threshold probabilities ranged from 2–18% for predicting two-year MACCEs risk and 2–95% for predicting three-year MACCEs risk (**Figure 8**, panels A–B). The threshold probability ranges in the validation cohort were 2–15% for two-year MACCEs risk and 5–80% for three-year MACCEs risk (**Figure 8**, panels C–D).

**Figure 9:**
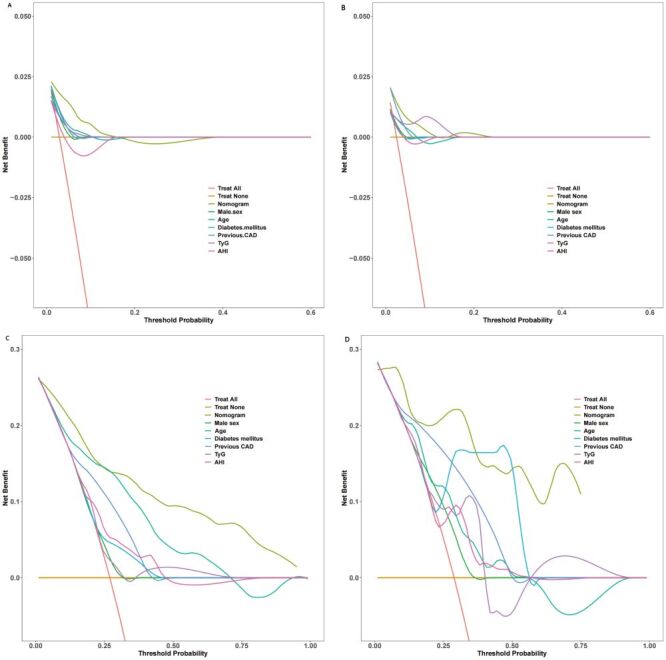
Net benefit rate of the prediction nomogram and separate models for the six independent variables (sex, age, diabetes mellitus, previous coronary artery disease, triglyceride-glucose index, and apnea-hypopnea index) in the development and validation cohorts. **Panel A.** Prediction of two-year MACCEs risk for the development cohort. **Panel B.** Prediction of two-year MACCEs risk for the validation cohort. **Panel C.** Prediction of three-year MACCEs risk for the development cohort. **Panel D.** Prediction of three-year MACCEs risk for the validation cohort. MACCEs – major adverse cardio and cerebrovascular events

**Figure 10:**
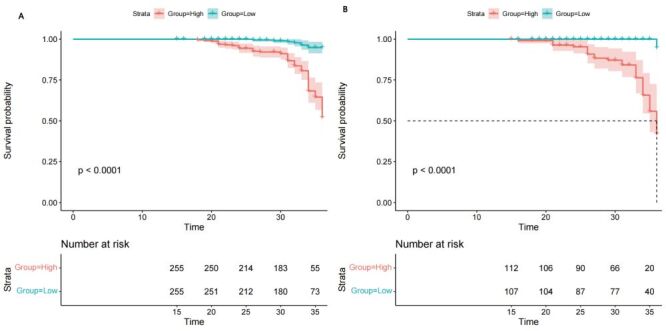
Cumulative MACCE-free survival stratified according to MACCE risk based on the median nomogram score. **Panel A.** Development cohort. **Panel B.** Validation cohort. MACEE – major adverse cardio and cerebrovascular events

Additionally, we conducted a decision curve analysis to determine if the predictive model offered more overall benefit compared to the individual variables. The prediction nomogram demonstrated a higher net benefit rate than the individual models for the six independent variables (sex, age, diabetes mellitus, previous CAD, TyG index, and AHI) in both the development and validation cohorts (**Figure 9**, panels A–D). Additionally, the median risk score from the nomogram was utilised to categorise patients into low- and high-risk groups in both the development and validation cohorts (**Figure 10**, panels A–B). The results of the Kaplan-Meier survival analysis revealed that individuals in the high-risk groups exhibited a higher incidence of MACCEs than those in the low-risk groups across both cohorts.

## DISCUSSION

In the present study, we used data from a large population of Chinese patients newly diagnosed with OSAS who were treated using standard treatment regimens to develop a prognostic model for cardiovascular disease risk based on six common clinical variables at baseline (sex, age, diabetes mellitus, previous CAD, TyG index, and AHI), encompassing multiple data modalities. With this model, we demonstrated that longitudinal prediction of prognosis at two and three years after initial diagnosis of OSAS substantially benefited from the integration of multimodal data compared with the use of unimodal data in our Chinese population. In summary, our research findings suggest that our forecasting chart can be a reliable and simple tool to help identify OSAS patients at a high risk of MACCEs in primary health care facilities in China, which can guide treatment choices to enhance outcomes for these individuals. Additionally, our results indicate that our nomogram may be helpful in the design and implementation of clinical trials that evaluate individualised clinical decision-making for OSAS patients. Appropriate and timely risk stratification of OSAS patients facilitates the implementation of targeted preventive measures, such as lifestyle modifications, optimised management of risk factors and closer monitoring. These interventions have the potential to reduce the incidence of costly MACCEs significantly.

Up to one billion middle-aged people have OSAS worldwide, and a marked increase in the number of OSAS patients has been observed with the expansion of the obesity epidemic [1–4]. However, a significant proportion of patients with OSAS remain undiagnosed, and those with moderate or severe OSAS are at high risk for poor outcomes [1–4,22,23]. With the prevalence of OSAS in our population, public awareness of the importance of controlling OSAS must be achieved. With low OSAS awareness, inadequate treatment and/or medication adherence contribute to poor prevention and control of OSAS among Chinese adults [1]. Conversely, early detection, precise preventative measures, and risk stratification lead to better OSAS patient prognosis and decrease the risk of MACCEs [7,17]. Evidence-based risk stratification can empower doctors to make appropriate decisions, give patients more control over their health, and give payers and policymakers confidence to invest in proper solutions [12,21]. A previous study provided clinical evidence that longitudinal prediction of risk of cardiovascular events in OSAS patients benefits substantially from integrating multimodal data over-relying on unimodal data [15]. The nomogram developed in that study included patient demographic and clinical characteristics with physiologic indices to provide greater accuracy for predicting cardiovascular outcomes. However, well-established biomarker data and information about CPAP adherence were unavailable [15]. Moreover, the study lacked a validation group and did not assess the clinical applicability of the nomogram. Importantly, a global perspective on risk prediction models has rarely been presented in clinical guidelines for the development and application of risk assessment. Given the heavy burden inflicted by the high prevalence of OSAS in China currently, our study indicates that a risk nomogram that incorporates conventional clinical data, a biomarker (TyG index), and sleep monitoring data (OSAS severity based on AHI) offers a simple yet valid tool that doctors can utilise to easily assess the risk of MACCEs in patients with initial diagnosis OSAS. Including routine clinical variables in our model ensures their availability across diverse health care systems worldwide, thereby supporting the applicability of our model beyond the Chinese population. This universality underscores the feasibility of implementation, provided that basic clinical data collection practices are established within the respective health care settings. While the predictors are universally accessible, the prevalence and impact of certain predictors may exhibit variations across different populations due to genetic, environmental, and lifestyle factors. To adapt our model for utilisation in diverse populations, it would be imperative to conduct population-specific recalibration by adjusting the weight assigned to each predictor based on its relative importance or prevalence within the new population. Furthermore, our prognostic model sets a benchmark for future research in OSAS and cardiovascular disease. It highlights the importance of considering various variables in risk prediction models, including those specific to OSAS. Future studies can build upon our approach, integrating additional biomarkers or clinical variables to refine risk prediction and patient management further.

Accumulating evidence and our data support the notion that OSAS and cardiovascular and cerebrovascular diseases have shared risk factors [7,24,25]. Consistent with previous research [24,25], our study displayed that patients with OSAS who are older, have diabetes, and have a history of CAD are at a higher risk of developing MACCEs. Also, assessment of the severity of OSAS through the AHI in our model is consistent with previous research indicating that a higher severity of OSAS often corresponds to a poorer prognosis for patients [6,15,26]. Including the AHI in the visual risk assessment tool probably boosted the tool’s additional prognostic value and its capacity to improve clinical decision-making. Currently, insulin resistance (IR) plays a pivotal role in the diagnosis and prognosis of OSAS and adverse cardiovascular events, enabling triage decisions in the high-risk care of the OSAS patient [27,28]. Pathophysiologically, the process and evolution of OSAS involve multiple pathophysiological mechanisms, among which IR is significant [29]. Studies have shown an increased incidence of IR in OSAS patients and that the degree of IR is positively correlated with the severity of OSAS [27–29]. IR affects not only glucose but also lipid metabolism and blood pressure regulation, exacerbating the pathophysiological process of OSAS and ultimately leading to a poorer prognosis [27–32]. Accumulating evidence supports that the TyG index is a reliable marker of IR [19,33,34]. Additionally, previous literature has shown that the TyG index can independently predict negative results in individuals with OSAS [10,28,35,36]. Our data exhibited that the TyG index enhanced the predictive accuracy of our nomogram for MACCEs, which is important for patients with OSAS. Notably, our model emphasises the necessity of moving beyond solely focusing on the AHI and instead advocates for adopting a comprehensive, patient-centred risk assessment approach to manage OSAS effectively. Indeed, successful OSAS management necessitates thorough consideration of various factors, including patient symptoms, comorbidities, and biomarkers. Moreover, the accurate prediction of cardiovascular disease risk by our model enables digital health intervention providers to customise treatment strategies based on each patient’s risk profile [36,37]. For instance, patients identified as high-risk may receive more comprehensive management of OSAS and associated cardiovascular risk factors, potentially including earlier initiation of CPAP therapy, more aggressive control of blood glucose and dyslipidemia, and closer monitoring for the development of cardiovascular complications. Integrating our prognostic model into clinical practice can support clinicians in making informed decisions regarding prioritising resources and interventions. This is particularly relevant in settings where health care resources are limited, and there is a need to identify patients most likely to benefit from specific treatment modalities. To enhance the utility and accessibility of our predictive model, we propose developing a web-based application. This platform will empower health care professionals to input patient-specific information and automatically compute the risk probabilities associated with the condition of interest. Upon entering the patient’s clinical variables into the application, the predictive model will promptly calculate and display real-time risk probability assessment. This instantaneous evaluation will assist clinicians in making well-informed decisions regarding patient care and management.

This study has several limitations. First, selection bias could not be avoided because this was a retrospective study. A prospective clinical study is needed to gather more substantial clinical evidence, and the results are then used to enhance the prediction model. Incorporating multicenter studies involving institutions from diverse geographical locations and health care systems would encompass a broader range of patient demographics. This is crucial for evaluating the predictive model’s performance across different ethnicities, lifestyles, and genetic backgrounds. This approach enhances the generalisability and applicability of the study findings. Second, the study population was treated at a single centre, which may reduce the generalisability of our findings. Third, as external validation data were not obtained, our study included only internal validation. Fourth, assuming that all predictor levels will remain unchanged throughout the follow-up period is overly simplistic. The trajectories of these predictors, such as lifestyle modification or OSAS progression over time, were unknown for our patients and could not be evaluated at baseline when the risk prediction was initially made. Further, the potential impact of emerging technologies, such as machine learning algorithms, may enhance the accuracy and relevance of existing predictive models. Future research will explore applying advanced machine learning techniques, such as deep learning, ensemble methods, and natural language processing, to enhance the model’s performance. These sophisticated methodologies are capable of effectively handling complex interactions and nonlinear relationships compared to traditional statistical models. Machine learning also provides tools for identifying and mitigating biases in predictive models. Fairness algorithms will be incorporated in future iterations of our model to ensure equitable predictions that do not inadvertently disadvantage any specific patient group. Finally, due to the retrospective nature of our study, we encountered certain limitations in acquiring comprehensive information regarding CPAP usage, patient adherence, and weight fluctuations. The heterogeneity in the types of respiratory devices employed by the patients presents additional obstacles in standardising the parameters.

## CONCLUSIONS

This research presents a novel predictive model based on various data sources to provide Chinese patients and their health care providers with a numerical risk assessment for MACCEs when diagnosing OSAS patients receiving standard treatments. This model provides a more intuitive and robust scientific tool for the predictive, preventive, personalised, and participatory care of these patients. Future research should prioritise the development of more efficient methodologies to integrate data, establish optimal practices for evaluating model effectiveness across diverse data types, and conduct prospective studies with large sample sizes to validate the clinical utility of these models.

## Additional material


Online Supplementary Document
