# Supplementary material for: Multimodal data integration for enhanced longitudinal prediction for cardiac and cerebrovascular events following initial diagnosis of obstructive sleep apnea syndrome
Source: J Glob Health. 2024 May 17;14:04103. doi: 10.7189/jgh.14.04103 (PMC11100360; doi:10.7189/jgh.14.04103)

Table S1. Inclusion and exclusion criteria.

**Inclusion criteria**
Initial Diagnosis of obstructive sleep apnea syndrome
Successful polysomnography screening
Follow-up was completed.
**Exclusion criteria**
Lack of sleep monitoring
Significant sleep disorders other than obstructive sleep apnea syndrome, including central sleep apnea, obesity hypoventilation syndrome, and insomnia
Previous diagnosis of or treatment for obstructive sleep apnea syndrome
Acute coronary syndrome
Acute cardiac failure
Implantation of pacemaker
Depressed left ventricular systolic function (ejection fraction <30%)
Malignant tumor
Severe aortic stenosis
Dilated cardiomyopathy
Rheumatic heart disease
Cor pulmonale
Myocarditis or cardiomyopathy
Infectious or severe liver or kidney disease
Lacked data on fasting triglyceride (TG) and fasting blood-glucose (FBG)
Patients without the results of a sleep monitoring study
Poor compliance to treatment

Lost to follow-up.

Figures S1. Study flow diagram.

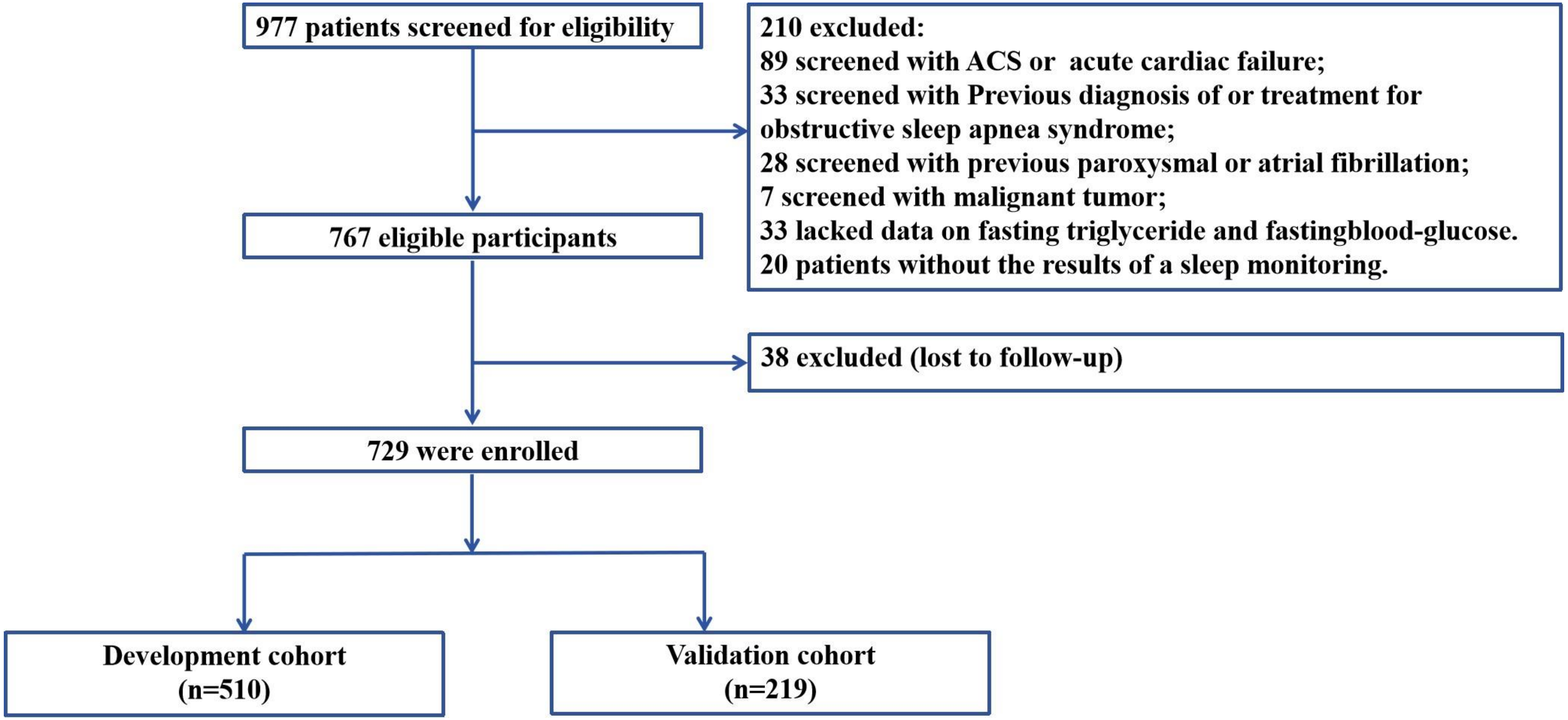

Supplement: Online Supplementary Document [file jogh-14-04103-s001.pdf]
